# An exploration of barriers to access to healthcare in Hancock County, Tennessee: A qualitative study

**DOI:** 10.1111/hex.14074

**Published:** 2024-05-20

**Authors:** Christine Crudo Blackburn, Tasmiah Nuzhath

**Affiliations:** ^1^ Department of Health Policy and Management, School of Public Health Texas A&M University College Station Texas USA; ^2^ Department of Global Health and Population, T.H. Chan School of Public Health Harvard University Cambridge Massachusetts USA

**Keywords:** conceptual framework of access to healthcare, emergency care, healthcare access, remote health, rural heath

## Abstract

**Objective:**

Explore barriers to healthcare access in Hancock County, Tennessee using a conceptual framework for access to healthcare.

**Methods:**

We collected data from 30 participants in Hancock County during 1 week in April 2023 using a combination of network and purposive sampling. We analyzed the data using thematic analysis and the conceptual framework of healthcare access.

**Results:**

All dimensions of the conceptual framework of healthcare access presented barriers to healthcare access for participants of the study. A lack of acceptability of local healthcare among participants manifested in a perceived lack of availability of healthcare. This resulted in participants travelling or considering it necessary to travel long distances for care, even in a life‐threatening emergency, despite the local availability of a hospital with an emergency department.

**Conclusions:**

A lack of acceptability can create healthcare access barriers similar to a lack of availability of healthcare facilities.

**Patient or Public Contribution:**

The research team met several times with the leader of a local community organization to discuss this research in Hancock County. These conversations helped to inform the study design and provided necessary background to conduct in‐depth interviews. Members of the community organization helped identify individuals to interview and provide access to Remote Area Medical clinic patients. The research team discussed the final themes with the primary community collaborator.

## 1 INTRODUCTION

Barriers to healthcare access contribute to health disparities between rural and urban communities that are socioeconomically and environmentally determined.^1^ Rural residents frequently have worse health outcomes, including higher rates of illness, injury, disability and early death.^2^ Rural communities also have numerous resource, financial and cultural constraints to healthcare access compared to urban communities.^3^ Resource constraints such as a shortage of trained physicians and nurses in rural areas to provide high‐quality care require people to travel further for care, thus contributing to overall health disparities compared to urban communities.^3^, ^4^, ^5^

One of the biggest challenges for both rural and urban Americans is healthcare affordability. Approximately 10% of all adults in the US report affordability‐related barriers to accessing healthcare^6^, ^7^ despite the implementation of the Affordable Care Act (ACA), which has improved access and affordability of care.^8^, ^9^, ^10^ Since ACA, overall uninsured rates have declined in the United States. However, those living in rural areas still have higher overall uninsured rates than those in urban areas.^11^, ^12^ Additionally, evidence shows that uninsured rates increase with a degree of rurality.^13^

Rural residents also frequently have a difficult time physically accessing healthcare services. Previous studies have found that transportation is another barrier to healthcare access.^14^, ^15^, ^16^, ^17^, ^18^, ^19^ A lack of transportation can lead to delayed care, missed or delayed medication usage and poorer health outcomes.^20^ On the other hand, direct access to transportation increases healthcare utilization for rural residents in the United States.^21^

Healthcare access has been conceptualized in numerous ways.^22^, ^23^, ^24^, ^25^, ^26^, ^27^, ^28^ Penchansky and Thomas,^24^ in their Concept of Access, discussed healthcare access as requiring the five As: availability, accessibility, accommodation, affordability and acceptability. Within this framework, healthcare must be physically available, in a geographic location that is reachable, in a structure the individual can navigate, at a cost they can afford and with a provider who they feel comfortable with. While this framework identifies vital barriers and facilitators for healthcare access, it ignores contextual and personal facilitators and barriers.

Contextual and individual dimensions of healthcare access have been shown to influence an individual's healthcare seeking behaviour. Studies examining the factors influencing perceived quality of care found that patients were less willing to go to a medical facility where they felt that the providers did not care about them or treated them poorly.^29^, ^30^ The relationship between the patient and the nurses, in particular, plays an important role in the patient's perception of the quality of care they received.^30^, ^31^, ^32^ Rural residents expressed higher levels of frustration with inefficiencies in the healthcare system^33^ and studies have found that wait times in a healthcare setting can impact how patients perceive the quality of the care they received.^34^ There is a relationship, however, between hospital performance ratings and patients' satisfaction ratings,^35^ suggesting that hospitals with more resources and highly trained personnel are more likely to provide patients with the feeling that they received high‐quality care.

Other studies^36^, ^37^ found that there was a large gap between the perceived quality of care that providers believed they were providing and the way that patients viewed the quality of care they received. Patients consistently viewed the quality of care they received as lower than how that care was perceived by the providers. This identifies a disconnect between how providers believe they are meeting the needs of their patients and how patients perceive their own needs. A disconnect between provider perceptions of care and patient perceptions of care is important because patients who have poor or negative experiences with their healthcare providers are less likely to see these providers in the future.^29^ Patients' level of satisfaction with healthcare is crucial as it affects the patient's healthcare‐seeking behaviour, which can influence their perceived health status.^38^

Andersen and Davidson^27^ address many of these shortcomings in their framework of healthcare access. This framework incorporates contextual factors including the influence of neighbourhoods and local communities, as well as individual factors such as demographic characteristics and how individuals respond to an illness or injury. The framework also addresses shortcomings in Penchansky and Thomas'^24^ concept of access by incorporating health behaviours.

In addition to the contributions by Andersen and Davidson,^27^ Levesque et al.^28^ provide critical framing for healthcare access by incorporating dimensions of ability into their expansion of Penchansky and Thomas'^24^ concept of access. The dimensions of ability include the ability to perceive, seek, reach, pay and engage. Like Andersen and Davidson's^27^ framework, Levesque et al.^28^ recognize that healthcare access has both a ‘supply side’ and a ‘demand side’. The dimensions of ability provide a framework for capturing the demand side by examining individual characteristics (like Andersen and Davidson) of culture, gender, health insurance, health literacy and other measures. Taken together, these frameworks seek to capture the nuanced and multidimensional nature of healthcare access.

Addressing barriers to healthcare access is important as they have been shown to influence an individual's healthcare‐seeking behaviour. In vulnerable communities, addressing healthcare access barriers will require identifying and intervening in the dimensions of healthcare access and social determinants of health.^39^ In this study, we explored the barriers to healthcare access in Hancock County, Tennessee using Levesque et al.'s^28^ conceptual framework of access to healthcare.

### 1.1 Setting

Hancock County was selected as the location for the study because it is designated by the US Department of Health and Human Services as a Medically Underserved Area, but Sneedville is home to a not‐for‐profit Critical Access Hospital. This hospital has a 24/7 emergency departments and provides a variety of diagnostic services.^40^ Hancock County also has a Rural Health Clinic, which provides annual physicals, primary care, immunizations, women's health services, diabetes management and nutrition counselling, and the Sneedville Medical Center, which provides basic primary and preventative care services. Additionally, the uninsured rate in Hancock County is estimated to be 11.6%,^41^ which is just slightly higher than the state average of 10%.

Despite these services and insurance rates that are similar to the state average, Hancock County ranks last out of the 95 counties in Tennessee for length of life and 86th in measure of clinical care, such as access to healthcare and quality of healthcare.^40^ The county has high levels of obesity and physical inactivity, as well as mortality rates for heart disease, cancer, and diabetes that are almost double the state average.^42^ Hancock County residents also have higher mortality rates from stroke compared to the state average and lower overall life expectancy.^43^

## 2 METHODS

### 2.1 Conceptual framework

**Figure 1:**
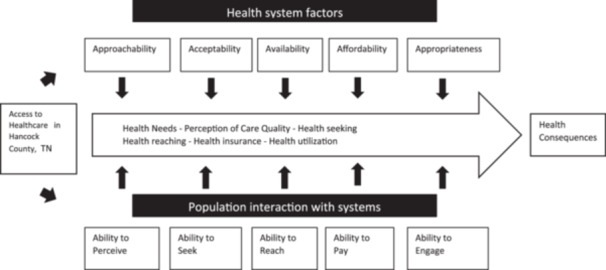
Guiding conceptual framework for access to healthcare in Hancock County, Tennessee.

Our study utilized the Conceptual Framework of Access to Healthcare developed by Levesque et al.^28^ The dimensions of Levesque et al.'s^28^ framework include both health services dimensions and individual abilities dimensions. The health services dimensions are: (1) approachability, (2) acceptability, (3) availability and accommodation, (4) affordability and (5) appropriateness. These health services dimensions are then matched with individual ability dimensions, which include: (1) ability to perceive, (2) ability to seek, (3) ability to reach, (4) ability to pay and (5) ability to engage. In Levesque et al.'s^28^ framework, approachability examines transparency, outreach, information and screening, while the ability to perceive examines health literacy, health beliefs, trust and expectations. Our framework, adapted from Levesque et al.,^28^ is shown in Figure 1.

### 2.2 Research team characteristics

Both members of the research team conducted interviews during this study. One team member (C. C. B.) is a White female in her 30s. C. C. B. has extensive experience conducting qualitative, interview‐based research in various settings, including rural and remote communities. The other research team member (T. N.) is a brown female in her late 20s. T. N. has previous experience in working with rural communities and conducting field interviews outside of the United States. As this was T. N.'s first field interview in the United States, TN was trained in qualitative methods by CCB before the initiation of this study. It is possible that the research team status as ‘outsiders’ affected the transparency of interview communication, but it is also possible that the research team gender and age reduced traditional power dynamics in researcher/participant relationships and reduced the impact of this bias.

### 2.3 Interview context

Interviews were conducted in various locations throughout Sneedville, Tennessee. Eight participants were interviewed at the local community centre. All interviews were conducted in rooms away from public areas of the community centre to protect participant privacy. Two of the interviews were conducted downtown. In both cases, the businesses were empty enough to provide the researcher and participant with privacy and ensure the conversation was not overheard. One interview was conducted in a private office at the local volunteer rescue fire station and one in a private office at the county emergency medical services station. Interviews were also conducted at a repurposed former school outside the town of Sneedville. The final interviews were conducted in the parking lot of a local elementary school, where people were waiting to be seen at the Remote Area Medical (RAM) pop‐up clinic. In these cases, the interviewing member of the research team stood outside the participant's car, and the participant either remained in their vehicle with the window down or stepped out of the car to talk with the research team member. Interviews with participants waiting for the RAM clinic took place between 9:00 PM and 3:00 AM, as they had to wait in the parking lot overnight to guarantee care in the morning when the clinic opened.

### 2.4 Sample size

Thirty individuals were interviewed for this study over 1 week in April 2023. The study design called for a minimum of 20 interviews and a maximum of 40 interviews, based on the general consensus in the literature that data saturation can be achieved with 20–40 interviews.^44^, ^45^ Since multiple trips were not feasible, we relied on this literature to choose sample size rather than conducting an iterative data collection and analysis process until saturation was reached.

We debriefed after each interview and determined after 30 interviews that data saturation had likely been reached. Data saturation was later confirmed during analysis.

### 2.5 Recruitment and sampling

Participants were recruited through network sampling and purposive sampling. Our community partner reached out to individuals in the community that she believed could meet our purposive sampling approach based on the three meetings we had with her before initiating the study. She organized interviews at the community centre and spoke to participants beforehand to help facilitate our interview process. She also coordinated with RAM officials to allow us to interview individuals waiting in the parking lot for care.

We targeted low‐income and older individuals in the community, as well as emergency responders, with the goal of understanding the dimensions of access to healthcare described in our conceptual framework. We arranged our travel to Tennessee at a time when the RAM clinic would be there to allow us to interview individuals that lived outside the town of Sneedville. We also learned that those attending the RAM clinic likely had issues accessing healthcare and would be able to speak directly to the primary barriers accessing care in the county.

Since individuals not interviewed at the RAM clinic were recruited through network sampling, only four individuals declined to participate in the study through this recruiting method. More than half of the individuals waiting for the RAM clinic declined to participate in the study. Participants were compensated with a $15 gift card to Subway. The demographics of the study participants are outlined in Table 1.

**Table 1:** Participant demographics.

Characteristic	Number of participants	Percentage of sample
Sex		
Female	22	73
Male	8	27
Race/Ethnicity		
White	29	97
Hispanic/Latino	1	3
Age		
18–29	8	27
30–39	3	10
40–49	6	20
50–59	4	13
60+	10	33

### 2.6 Participant experience and consent process

Those recruited through network sampling were aware of the interview before the interview date. They were provided with a detailed description of the study and the informed consent process at the time of the interview. Participants were given time to read through the informed consent document. Once an individual agreed to participate in the study, they signed the informed consent document. The research team member collected the informed consent, told them when the recording began and reminded them that their participation was voluntary, and then began the interview.

For interviews conducted at the RAM clinic, potential participants were approached at their vehicles in the parking lot of the elementary school. They had been informed by RAM security staff that we were going to be asking for participants for our research project. Once approached, we provided study details and informed consent. We asked them if they would like to participate in the study. If the potential participant said no, we thanked them for their time and moved to the next vehicle. If they said yes, we followed the same procedure that we used with the participants found through network sampling.

The average length of time per interview was 30 min. All interviews for this project were conducted by the two members of the research team (C. C. B. and T. N.). Interviews were unstructured, though we ensured that the questions addressed the key dimensions of the conceptual framework. Examples of constructs and questions can be found in Table 2.

**Table 2:** Interview guiding constructs and example questions.

Construct	Example question
Approachability	How does trust in your healthcare provider influence your decision whether to go to the doctor when you are sick or injured?
Ability to perceive	
Acceptability	How does your healthcare provider's behaviour towards you influence your decision whether to go to the doctor when you are sick or injured?
Ability to seek	
Availability/accommodation	How does the distance between here and the hospital/doctor's office affect your decisions about whether and when to get medical care?
Ability to reach	
Affordability	Do you have concerns about the cost of healthcare? How does this affect your decision to see a doctor when you are sick or injured?
Ability to pay	
Appropriateness	When you do see a doctor, do they give you information in simple language and terms you can understand?
Ability to engage	

### 2.7 Analysis

Following completion of the interviews, the audio data was transcribed both by hand and using the transcription service Rev^©^. The transcripts were quality checked by one research team member (C. C. B.) to ensure they were transcribed verbatim from the audio data. Thematic analysis was conducted using MAXQDA^©^ 2022 software. We utilized a deductive orientation to our coding with a focus on coding the data based on Levesque et al.'s^28^ conceptual framework of healthcare access. Within our deductive orientation, we applied semantic coding, which captures the explicit expressions of the participants.^46^ Within this coding approach, the codes ‘stay close to the language of participants or the overt meanings of the data’.^46^ ^,p.57^ The focus of the first round of coding was to create codes that described exactly what the participant had said about healthcare.

During the second round of coding, codes that were not mentioned frequently enough to rise to the level of themes or did not fall within the conceptual framework were eliminated and remaining codes were grouped into the framework dimensions. During the second round of coding, we applied a latent coding approach, which focuses on implicit meanings within the data.^46^ Frequency of code appearance was determined by the number of participants who mentioned the concept captured in the code rather than the number of times that concept was coded. This ensured that final codes would represent themes across participants.

During a third round of coding, again utilizing latent coding, final themes were identified within the structure of Levesque et al.'s^28^ conceptual framework. While the final codes were checked against the initial codes to make sure that participant intention was not lost in the analysis and consolidation process, we did not use any method of participant validation.

## 3 RESULTS

‘I love living here, but the healthcare system is what kills a lot of these people’, a volunteer firefighter in Hancock County, told us. She and the other 29 individuals we interviewed for this study expressed concerns about healthcare access in Hancock County. Those concerns were identified and grouped using the conceptual framework of healthcare access and include approachability/ability to perceive; acceptability/ability to seek; availability/ability to reach; affordability/ability to pay and appropriateness/ability to engage.

### 3.1 Ability to perceive

#### 3.1.1 Lack of trust in healthcare providers—‘… they don't trust the doctors, or they don't feel confident’

Within Levesque et al.'s^28^ conceptual framework of healthcare access, the ability to perceive contains the elements of health literacy, health beliefs, trust and expectations. This dimension, which is the first of the five dimensions of abilities and aligned with the first health service dimension of approachability, frames the way that individuals perceive their healthcare providers, an important component of which is how much they trust them. Through our analysis, we found that mistrust of healthcare providers was common, with many participants expressing a general mistrust of medical professionals in the region. They had low expectations of what the providers could do for them, and they often felt they should deal with their health issues on their own. As one participant expressed,

Yeah, there's, I mean, culturally, there's a lot of distrust for outside doctors. They'll go to the people who were from here, went out, came back, even though they aren't being treated well. And then you have some [doctors] that … [say] I'm going to go serve an underserved population. They go in, and they're not from there, so you don't trust them. That's just culture.

All participants were rural residents and placed an emphasis on familiarity. This was not true only of healthcare providers, but more broadly of all community members. Participants who had moved to the community or whose parents had moved to the community, expressed that they were sometimes treated as outsiders because their family hadn't been there for generations. This broad distrust of outsiders made participants distrustful of outside doctors and nurses who came to serve the community.

Another participant explained that their lack of trust went beyond the medical provider's status as an outsider. Distrust also stemmed from the way they were treated and their perceived quality of care. This participate discussed an experience that led to the distrust he currently holds in local medical providers saying,

[Referring to the local emergency room] It's horrible. That's where, this is probably 4 or 5 years ago when I was having my surgeries and stuff and they, honestly, they just treated me like a guinea pig up there. They had no clue what they were doing.

Feelings of distrust were common among participants, as were comments that the one trusted doctor had passed away during COVID‐19. People spoke fondly of him, felt that he had treated them with respect, and noted that he frequently made house calls at all hours of the day and night. Since his passing, most participants felt there was no medical provider they could rely on.

### 3.2 Acceptability/ability to seek

#### 3.2.1 The local hospital is not considered an option for healthcare—‘I will crawl, roll, slither to my car and drive myself to Kingsport’

Most study participants didn't believe that the local hospital could provide sufficient care. They suggested that it would be better to drive themselves to a different hospital, even in a life‐threatening emergency, as exemplified by the title quote. One participant, when asked if they used the local hospital, stated, ‘I try my best just not to fool with this hospital here’. Many participants shared this sentiment, as another participant confirmed by saying:

I mean, the emergency room up here is a joke. It's just, everybody knows it. Nobody uses it. I would rather take, um, if it was really like an emergency, I would feel more comfortable taking my time. Like have my wife drive me to Morristown as [opposed] to go up there, waste time, and for them to send me somewhere else. If it was really, you know, life sensitive, it would be better just to go on [to another hospital].

The assertion that participants wouldn't be willing to use the emergency room even in life‐threatening scenarios demonstrated the extent to which they lacked acceptance for the hospital. Another participant confirmed,

If I have to go to the emergency [room], I drive to anywhere other than [that hospital], cuz, I mean, they can't do anything.

Many of the participants had used the hospital previously and their lack of acceptance was a result of the negative experiences they had in the past. Many felt that the quality of care had been poor, that they had been treated badly, or that the hospital had simply sent them along to another hospital. One participant summarized this sentiment, saying:

They transfer everybody. They transfer every single person that has something wrong with them.

### 3.3 Availability/ability to reach

#### 3.3.1 It is necessary to travel to get needed healthcare—‘You're going to have to travel’

Many participants spoke of the effects of the lack of availability of local healthcare services. Almost every participant said that they had to travel to receive any kind of care. One participant stated, ‘There's just a lack of healthcare … there's nothing here’. While another said, ‘As far as medical, as far as in Sneedville, you can't get very much done’. Most participants stated that they travelled to Morristown, Rogersville, or Kingsport for both basic and specialty health services. Very few participants used either of the local medical centers.

For some, Hancock County truly doesn't provide the specialty care that they need, as one participant described,

I have to drive two hours away just to go see [my doctor]. I go every week, just to go there to get [treated], I'm taking Hep C treatments and stuff. And I have to go up there every week, 2 hours [each way].

The necessity of travel for specialized treatment, dentists, and eye doctors was very common among participants. For individuals who had to travel for care, transportation was a primary issue. One participant stated,

There's transportation companies here … I think there's two of them, but I think they say you pay like $40 to get a ride over the mountain and then you have to call them to get a ride home and you might have to wait two, three hours for them to come get you.

There was a general feeling among participants that the transportation companies were unreliable or too inconvenient to be useful. Numerous participants mentioned that they often had to wait hours for the transportation company to pick them up after their appointment. Other participants said the transportation company would sometimes cancel their reservation, so they would miss their appointment. In either case, most participants had negative experiences with the transportation company and preferred to rely on a friend or family member for travel to appointments.

For participants with substance abuse treatment needs, the lack of nearby services could be deadly,

There's no NA meetings here. AA meetings, nothing like that. I mean, there's no kind of support at all. And like for the medication, the Suboxone treatment stuff, I mean, it's been a lifesaver for me, but people around here, you know, if they ain't got vehicles or, you know, a lot of people just ain't got resources able to do it.

Participants who were in recovery from substance abuse expressed that they had lost many friends due to overdoses and that they believed that fewer people would have died had services been available locally. One participant stated,

You know, I've had within the last, like, last year, two years, at least 20 of my friends are dead from fentanyl.

This participant went on to say that he gets Narcan from his doctor, located several hours away, and hands them out to people in the community. He said that people with substance abuse disorders must look out for each other because services are too far away.

Whether the need to travel was based on a lack of acceptability of local healthcare services or a true lack of services available locally, most participants said some version of the following: ‘We don't have anybody that's going to help. There's nobody here to help’.

### 3.4 Affordability/ability to pay

#### 3.4.1 Healthcare delay and avoidance because of inability to pay—‘The healthcare system is unaffordable’

All the individuals that we interviewed in the school parking lot were waiting for the RAM clinic to open. Many said they had travelled a long distance to access the free care the RAM clinic provided. Most participants, not just those interviewed while waiting for the RAM clinic, delayed care or avoided care because of cost. One participant stated:

And you know, there's a lot of people, even like myself, every time I'll get sick, they'll do their own little something. I ain't going there [doctor's office] and paying them no $2,000. I'll just take a cold medicine or whatever, [and] wait it out

Self‐treatment was a common way that participants at the RAM clinic dealt with medical needs because they felt unable to access a doctor due to affordability. One participant, who was hoping to see a dentist at the RAM clinic spoke about his self‐treatment saying,

Unfortunately, how I normally deal with it is just massive amounts of painkillers until the nerve dies. And then you know I wait for the next tooth to die.

And while self‐treatment due to inability to afford care was common, equally common to choose not to seek care even when the participant felt that they really needed to see a doctor. One participant explained this saying,

Yesterday my blood pressure was high and I've had something going on like a cold and stuff. Can't get over it. Felt the need that I needed to go somewhere and didn't because I don't have insurance and can't afford to pay.

For many of the participants that we interviewed, paying for food and housing costs was a large enough challenge. They often felt that they had to pick and choose the bills they could pay and, often, healthcare was one of the things that got neglected. As explained by one participant, ‘I will take care of my healthcare when I've taken care of my food and utilities. And because it is so close to the edge, it's the last thing. It's one of the things you ignore’.

### 3.5 Appropriateness/ability to engage

#### 3.5.1 Poor interpersonal quality and lack of empowerment—‘I've been treated better by my vet than I have by my own doctors’.

Levesque et al.^28^ incorporates the elements of technical quality, interpersonal quality, adequacy and coordination and continuity under the dimension of appropriateness. Additionally, the paired ability, ability to engage, contains the elements of empowerment, information, adherence and caregiver support. Most participants in the study discussed the poor interpersonal quality of the care they received and the lack of empowerment they felt when interacting with medical professionals. Specifically, many individuals felt that their providers humiliated or embarrassed them. Interestingly, many of these humiliating experiences occurred when interacting with doctors that were not ‘outsiders’. During a discussion of poor treatment and humiliation by doctors, one participant commented on those doctors,

They were born, raised there, went away for school, came back to serve their people.

The participant was then asked for clarification purposes if those doctors treated people poorly, to which they responded, ‘yes,’ then went on to elaborate that since those individual's went off to school, they looked down on the people who had never left the community.

Many participants felt they were treated poorly when they tried to access local healthcare. Some of this poor treatment stemmed from their criminal or drug use history and some of it was the belief that they had been treated as ignorant by their healthcare providers. One participant explained their experience by saying:

Some would minimize the symptoms. Some would say, ‘well, it's just not that bad’. Then some would go on and do the, ‘well, you should quit Googling things’.

This participant expressed frustration and humiliation in their experiences with the local healthcare system. Many other participants had similar experiences of being treated like they were ignorant. Another participant said,

But I got treated like I was ignorant, and I was just looking things up and didn't know what I was talking about.

The participants who experienced this treatment all stated that they would not go back to the facilities that had mistreated them.

When discussing negative treatment that resulted from current or former criminal behaviour or drug use, one participant said, ‘They're willing to put you in handcuffs right there in the hospital. For one, it's embarrassing. For two, why would you put somebody in jail?’ Many participants mentioned that hospitals run patients’ names for warrants before they would provide them with care:

They won't go to the hospital because apparently now the hospitals run your name. So, if you have warrants, you get arrested right after you're released from the hospital. Sometimes you don't even get released before you're arrested.

A participant who had a particularly negative experience accessing care claimed that people were denied care because they had a substance abuse disorder or because they did not have insurance. She explained:

You show up there and then they don't, they're not going to help you. They'll turn you away. Over insurance, over the smallest thing, you know, and there's addicts out here that die everyday because of this situation. Because they know that, you know, they're going to get their name ran. They're going to not be treated equally as somebody who's got insurance. Somebody who's got money, you know. They should be helping people regardless of what their situation is. You know, you might be an addict, but at the same time, your life matters.

This claim and others regarding denial of treatment to the participants themselves, their friends, or their family members was both concerning and provided ample information to understand why most participants did not trust the local healthcare services or view them as an option for care. The individual who made the statement above said that she had to go to the University of Tennessee Medical Center in Knoxville, almost 2 h away, before she could find someone who would treat her despite being told she would die if she did not receive treatment.

## 4 DISCUSSION

Our findings highlight the barriers to healthcare access for individuals in Hancock County using the conceptual framework of healthcare access. Participants discussed the lack of ability to engage at their local hospital with a specific focus on the way that they were treated by healthcare providers there. Previous research has shown that feelings of discrimination in a healthcare setting can present a barrier to care, affect a patient's perception of the quality of care they receive, and can result in worse health outcomes for the patient experiencing discrimination^47^, ^48^, ^49^ Our study confirms the findings related to discrimination and patient perceptions of care quality and healthcare utilization.

Our findings add to the literature by detailing the stigma and discrimination experienced in rural Eastern Tennessee and the criminalization of those with substance abuse disorders in the healthcare setting. Many participants experienced stigma and discrimination in the healthcare setting due to their drug use or insurance status. Additionally, most participants expressed that healthcare providers in the county treated them like they were ignorant. Consistent discrimination and ill‐treatment in local healthcare facilities contributed to unwillingness to use those facilities, even in an emergency. While examining the health outcomes of individuals in the community is beyond the scope of this study, it is likely that healthcare avoidance and delays resulting from experiences of stigma and discrimination will result in poorer health for community members.

Feelings of discrimination and stigma in a healthcare setting speak to issues around the quality of care for the community of study. Several studies have examined the factors that influence a patient's perception of the quality of care they receive and found that patients were less willing to go to a medical facility where they felt that the providers did not care about them or treated them poorly.^29^, ^30^ Our findings confirm these previous findings, as we see that participants who felt discriminated against, stigmatized or treated as if they were ignorant in healthcare settings were unwilling to utilize those facilities in the future. Due to the extremely rural nature of the study community, perceptions of the quality of care they received and the subsequent unwillingness to utilize those facilities in the future due to past experiences, translates into avoidance of the nearest healthcare facilities.

Participants repeatedly said that there were no health services available locally or stated that the available services wouldn't help anyone. Their perceived lack of availability was created by a lack of acceptability because most participants viewed their healthcare access similarly to if the local hospital and clinics did not exist. Previous research has shown that a lack of physical access to healthcare requires individuals to travel for care, delays needed care, reduces chances of appropriate treatment and exacerbates chronic health conditions.^20^ Due to the previously discussed stigma in healthcare settings experienced by many participants, they perceive a lack of availability of health services. This perception results in travelling far distances to receive healthcare, despite the physical availability of a rural clinic and rural hospital. This finding adds to the existing literature and suggests that the construct of acceptability and availability are linked, and that availability should be examined through patient or resident perceptions rather than simple physical location only.

There is extensive research on the role of affordability as a barrier to healthcare access, particularly for rural and vulnerable communities.^50^, ^51^, ^52^, ^53^, ^54^ The lack of health insurance coverage and general lack of affordability of healthcare contributes to poorer health outcomes and higher mortality rates.^21^ Our findings of affordability/ability to pay in Hancock County support the findings of previous literature. Participants who lacked insurance or were underinsured discussed their concerns about their ability to pay for needed healthcare. Participants spoke vividly about the pain that they lived with because they were unable to access necessary healthcare due to costs. Our findings suggest that many individuals in Hancock County experience concerns about their ability to pay for health services. This concern leads to the avoidance of health services, which likely contributes to the high rates of morbidity in the region.

Participants in our study experienced barriers in approachability, acceptability, availability and accommodation, affordability and appropriateness on the healthcare side, as well as barriers in ability to perceive, ability to seek, ability to reach, ability to pay and ability to engage on the health‐seeking side. Thus, most participants experienced barriers in all the dimensions of healthcare access within Levesque et al.'s^28^ framework. The barriers they experience in these dimensions, such as discrimination, perceptions of quality of care, travelling far distances for care, and an inability to pay for medical care, influenced their utilization of healthcare services.

### 4.1 Limitations

Due to the qualitative nature of this study, it is not possible to generalize these findings to all rural communities. The findings of this study only reflect the experiences and opinions of the individuals within Hancock County. Those in rural communities in other parts of Tennessee or other areas of the rural United States may have different barriers to healthcare access. Additionally, network sampling led to skewedness in participant demographics, with more young participants compared to the average for the county. This skew towards younger participants likely affected our findings by overrepresenting concerns of this younger demographic. Despite these limitations, however, the findings have implications for healthcare access and should encourage future research to examine if acceptability influences perceptions of availability in other rural communities in the United States. Neglecting these perceptions may lead to an overestimation of rural health access in the United States.

Our study demonstrates the ways that the dimensions of healthcare access compound, creating larger barriers than any individual dimension produces on its own. While our study has limitations, its strength is in expanding our understanding of the interrelatedness of these dimensions. Notably, the lack of acceptability of local health services creates an artificial lack of availability of health services.

## 5 CONCLUSION

This study examined barriers to healthcare access in Hancock County, using the conceptual framework of healthcare access developed by Levesque et al.^28^ Our findings show that barriers within the acceptability dimension create an artificial barrier within the availability dimension. Acknowledging and examining the interrelatedness and compounding nature of the dimensions of healthcare access could provide a more accurate picture of healthcare access in the rural United States.

## AUTHOR CONTRIBUTIONS

**Christine Crudo Blackburn**: Conceptualization; investigation; writing—original draft; methodology; formal analysis. **Tasmiah Nuzhath**: Investigation; writing—review and editing; formal analysis.

## CONFLICT OF INTEREST STATEMENT

The authors declare no conflict of interest.

## ETHICS STATEMENT

This study was approved by the Sam Houston State University IRB (IRB#: IRB‐2023‐28) and the Texas A&M University IRB (IRB#: IRB2023‐0790M). All participants gave written informed consent and were reminded that their participation was voluntary.

## Data Availability

Data is available upon reasonable request from the corresponding author. The data are not publicly available due to privacy or ethical restrictions.
